# Triple Negative Breast Cancer: Updates on Classification and Treatment in 2021

**DOI:** 10.3390/cancers14051253

**Published:** 2022-02-28

**Authors:** Maroun Bou Zerdan, Tala Ghorayeb, Fares Saliba, Sabine Allam, Morgan Bou Zerdan, Marita Yaghi, Nadeem Bilani, Rola Jaafar, Zeina Nahleh

**Affiliations:** 1 Department of Hematology and Oncology, Maroone Cancer Center, Cleveland Clinic Florida, Weston, FL 33331, USA; bouzerdm@upstate.edu (M.B.Z.); yaghim@ccf.org (M.Y.); 2 Department of Internal Medicine, SUNY Upstate Medical University, Syracuse, NY 13210, USA; 3 Department of Obstetrics and Gynecology, McGovern Medical School, UTHealth Texas, Houston, TX 77030, USA; tala.ghorayeb@uth.tmc.edu; 4 Faculty of Medicine and Medical Sciences, Holy Spirit University of Kaslik (USEK), Jounieh 1200, Lebanon; fares.c.saliba@net.usek.edu.lb; 5 Faculty of Medicine, University of Balamand, Beirut 11 00 2807, Lebanon; sabine.allam@std.balamand.edu.lb; 6 Faculty of Medicine, American University of Beirut, Beirut 1107 2020, Lebanon; mjb25@mail.aub.edu; 7 Department of Internal Medicine, Icahn School of Medicine at Mount Sinai, New York, NY 10029, USA; nadeem.bilani@mountsinai.org; 8 Department of Surgery, Faculty of Medicine, American University of Beirut Medical Center, Beirut 11097 2020, Lebanon; rola.jaafar@gmail.com

**Keywords:** triple negative breast neoplasms, Poly (ADP-ribose) polymerase inhibitors, immune checkpoint inhibitors, immunoconjugates

## Abstract

**Simple Summary:**

Triple negative breast cancer (TNBC) represents 15 to 20% of all breast cancers in the United States. The main treatment option remains chemotherapy, despite limited efficacy. New biologic and targeted agents are increasingly emerging for the treatment of TNBC. Given the continuous advances in the field of TNBC, this review assesses the latest developments in basic characterization, subtyping, and treatment of TNBC, including novel drug developments with antibody-drug conjugates, immune checkpoint inhibitors, PARP inhibitors, and androgen receptor targeted agents.

**Abstract:**

Breast cancer (BC) is the most common malignancy affecting women. It is a highly heterogeneous disease broadly defined by the differential expression of cell surface receptors. In the United States, triple negative breast cancer (TNBC) represents 15 to 20% of all BC. When compared with other subtypes of BC, TNBC tends to present in younger women, and has a higher mortality rate of 40% in advanced stages within the first 5 years after diagnosis. TNBC has historically had limited treatment options when compared to other types of BC. The mainstay of treatment for TNBC remains cytotoxic chemotherapy despite the emergence of new biologic and targeted agents. Defining the specific tumor molecular profile including PDL-1 and androgen receptor testing is expanding treatment options in the clinical setting. Identifying more targetable, novel biomarkers that may better define therapeutic targets or prognostic markers is currently underway. TNBC nomenclature is expected to be updated in favor of other nomenclature which would help direct therapy, and further redefine TNBC’s heterogeneity. Given the continuous advances in the field of TNBC, this review assesses the latest developments in basic characterization, subtyping, and treatment of TNBC, including novel drug developments with antibody-drug conjugates, immune checkpoint inhibitors, PARP inhibitors and androgen receptor targeted agents. Future trials are necessary in the face of these innovations to further support the use of new therapies in TNBC and the detection of the appropriate biomarkers.

## 1. Introduction

Breast cancer (BC) is the most common malignancy affecting women. It is a highly heterogeneous disease, encompassing several BC molecular subtypes, broadly defined by the differential expression of cell surface receptors. Triple negative breast cancer (TNBC) refers to breast neoplasms that do not express estrogen receptor (ER), progesterone receptor (PR), or human epidermal growth factor receptor 2 (HER2) on their cell surface. In the United States, TNBC represents 15 to 20% of all BC [1]. BC common intrinsic molecular subtypes include Luminal A, Luminal B, and Her2 overexpressing, and basal cell tumors, further stratified into special subtypes [2]. Gene expression profiling analysis classifies TNBC as a subtype of basal-like BC, with a 56% overlap in gene expression profiles [3]. When compared with other subtypes of BC, TNBC tends to present in younger women, and has a higher mortality rate of 40% in advanced stages within the first 5 years after diagnosis [4,5]. Around 45% of patients diagnosed with advanced stage TNBC will develop distant metastasis to the brain and/or visceral organs, with a median survival time of 13.3 months [6]. Some reports also suggested a higher recurrence rate in TNBC, reaching as high as 25%. Specifically, residual micro-metastatic disease following neoadjuvant chemotherapy in TNBC is associated with an increased risk of tumor recurrence, with limited options for conventional postoperative adjuvant chemotherapy. As a result, there has been a significant and constant increase in the number of clinical trials targeting TNBC [7].

Given the continuous advances in the field of TNBC, this review assesses the latest developments in basic characterization, subtyping, and treatment of TNBC, including novel drug developments with antibody-drug conjugates, immune checkpoint inhibitors, PARP inhibitors, and androgen receptor targeted agents.

## 2. Triple-Negative Breast Cancer Molecular Subtyping

In 2011, Lehmann et al. categorized TNBC into six subtypes [8]: basal-like 1 (BL1), basal-like 2 (BL2), mesenchymal (M), mesenchymal stem-like (MSL), immunomodulatory (IM), and luminal androgen receptor (LAR), by performing gene expression profiling of tumor samples from 587 TNBC patients [9]. In 2015 Burstein et al. studied samples from 198 patients and suggested dividing TNBC into two major groups based on quantitative DNA expression, further categorized into four subtypes based on identified potential targets [10] including the LAR group, which expresses androgen receptors (AR) and cell-surface mucin receptors (MUC1)—this subtype alone forms group 1; the mesenchymal subtype (MES) which expresses growth factor receptors such as platelet-derived growth factor receptor-α [PDGFRα] and c-Kit receptor; the basal-like immunosuppressed (BLIS) subtype, which expresses the immunosuppressive molecule V-Set Domain Containing T-cell activation inhibitor 1 (VTCN1); and the basal-like immune-activated (BLIA) subtype, which exhibits activation of the signal transducer and activator of transcription (STAT). The three subtypes MES, BLIS, and BLIA formed group 2, as they had similar gene expression profiles.

**Figure 1:**
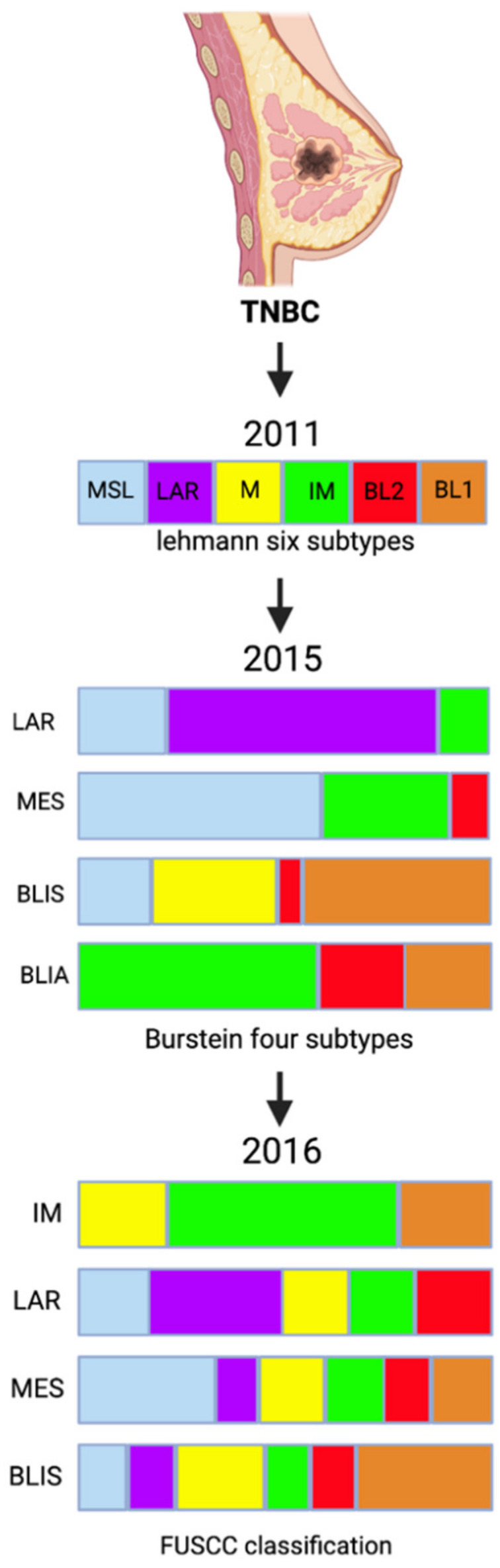
TNBC classification over the years.

In view of the emerging important role of long noncoding RNAs (lncRNAs) in cellular processes, a new classification incorporating both messenger RNA (mRNA) and lncRNA transcriptome profiles was suggested to help provide a better understanding of the heterogeneity of TNBC (Figure 1). In 2016, Liu et al. performed a categorization analysis of 165 TNBC samples that combined mRNA expression analysis and co-expression network analysis, aiming to identify interactions between mRNAs and lncRNAs [11]. They also investigated IM subtype genes, previously linked to the stimulation of T-cells and to innate and regular immune responses (Table 1) [6]. A strong association was identified between immune cell processes and TNBC tumorigenesis. IM subtype genes were identified to engage in regulating immune cells through their modulation of cytokine signaling, antigen processing, and immune cell signaling pathways involving T-cells, B-cells, chemokines and the nuclear factor-κB (NF-κB) [11]. Receptor-interacting protein 2 (RIP2) has been linked to chemoresistance of TNBC against paclitaxel. Jaafar et al. demonstrated that high expression of RIP2 correlated with a worse prognosis and a higher risk of recurrence since RIP2 lead to NF-κB activation, which contributed to higher expression of pro-survival proteins and cell survival [12]. Other genes, known to impact immune response—such as C-C motif chemokine teceptor-2 (CCR2), chemokine ligand 5 (CCL5), cluster of differentiation 1 (CD1C), C-X-C motif chemokine ligand 10 (CXCL10), C-X-C motif chemokine ligand 11 (CXCL11), and C-X-C motif chemokine ligand 13 (CXCL13)—were also expressed in the TNBC IM subtype, further confirming the role of immunity in TNBC IM tumorigenesis.

Conversely, tumorigenesis in the LAR subtype is closely related to hormonal regulation and activity, particularly the metabolism of androgen, chlorophyll, estrogen, and porphyrin, as well as the biosynthesis of hormones. LAR cells also show an increased expression of the peroxisome proliferator-activated receptor-γ (PPAR-γ). PPAR-γ is implicated in tumor cell proliferation, growth invasion, and phenotypic changes in differentiation status, but also correlates with quantitative androgen receptor expression, a defining feature of LAR [13]. Interestingly, despite lack of ER expression on its cell surface, LAR is clinically responsive to both anti-estrogen and anti-androgen therapy. This can be explained by the positive molecular activity of the estrogen receptor signaling pathway implicated in LAR tumorigenesis, despite LAR cells being ER-negative [10].

On the other hand, the MES subtype harbors a unique gene ontology, characterized by the interaction between extracellular matrix receptors, gap junction transmembrane channels, the transforming Growth Factor-β (TGF-β) signaling pathway, and growth factor-associated pathways, notably the adipokine pathway and ATP-binding cassette (ABC) transporters pathway [14].

Furthermore, the BLIS subtype is distinguished by a pathogenesis that strongly implicates cell cycle and resultant cell division processes in addition to DNA repair, replication, and regulation mechanisms. BLIS cells show increased quantitative expression of genes involved in cell proliferation such as the mitotic checkpoint serine/threonine-protein kinase budding uninhibited by benzimidazoles 1 (BUB1), and the protein coding genes centromere protein F (CENPF) and protein regulator of cytokinesis 1 (PRC1). This translates into a highly proliferative clinical nature of BLIS tumors [11], further allowed by the downregulation of immunologic processes specifically involving T-cell signaling, B-cell activation and dendritic cells chemotaxis. These molecular processes translate into shorter relapse-free survival (RFS) and increased recurrence rate on the clinical level, supporting previous findings by Burstein et al. [11].

Although progress in next generation sequencing has facilitated unraveling potentially actionable targets, not many findings have not been translated into daily clinical practice due to limited benefit from targeted therapy observed in clinical trials for unselected TNBC patients. The molecular subtyping enables the identification of molecularly homogenous groups with enrichment of certain genomic alterations. This paves the way for effective methods for drug development using subtype-specific clinical investigations. A precision medicine paradigm in the context of transcriptomic subtyping should be developed and fine-tuned for patients with TNBC.

## 3. Chemotherapy for Triple Negative Breast Cancer

TNBC has historically had limited treatment options when compared to other types of BC. The mainstay of treatment for TNBC remains cytotoxic chemotherapy, despite the emergence of new biologic and targeted agents. The therapeutic benefits of cytotoxic chemotherapy in TNBC are well established, with comprehensive data on the efficacy of chemotherapy in the neoadjuvant, adjuvant, and metastatic settings. Compared with hormone receptor-positive (HR+) BC, the use of chemotherapy regimens in the neoadjuvant treatment of TNBC has a significantly higher pathological response rate and can considerably ameliorate the prognosis of TNBC patients [15]. Nevertheless, TNBC carries an overall inferior prognosis despite its chemo-sensitivity [16]. The use of neoadjuvant systemic treatment (NST) in the early stages is becoming the standard of care in TNBCs and is associated with higher pathological complete response (pCR) rates (30–40%) as compared to other BC subtypes [17]. Patients who achieve pCR with primary therapy have improved survival outcomes [18]. As such, pCR is predictive of improved long-term outcomes for TNBC and is a reliable endpoint in clinical trials evaluating the efficacy of neoadjuvant chemotherapy.

Adriamycin, cyclophosphamide, and paclitaxel combinations are considered to be standard neoadjuvant chemotherapy regimen against TNBC and result in pCR rates of 35–45% [19]. The addition of platinum-based chemotherapy has been proposed. Despite improved short term pCR rates, long term outcomes remain unknown [20]. The systemic chemotherapy regimens options for TNBC recommended by National Comprehensive Cancer Network (NCCN) guidelines include the following: Docetaxel and Cyclophosphamide (TC), Taxel/Docetaxel, Adriamycin, and Cyclophosphamide (TAC), Adriamycin and Cyclophosphamide (AC), Cyclophosphamide, Methotrexate, and Fluorouracil (CMF), Cyclophosphamide, Adriamycin, and Fluorouracil (CAF), and Cyclophosphamide, Epirubicin, Fluorouracil and Paclitaxel/Docetaxel (CEF-T). These DNA damaging agents show increased activity in cancers with a germline BRCA mutation, as BRCA 1/2 proteins play an essential role in repairing DNA damage [21].

TNBC is also highly sensitive to platinum salts because a high proportion of these tumors exhibit BRCA-like status [20,22,23]. Two large, randomized trials—CALGB 40603/Alliance trial and GeparSixto—compared conventional chemotherapy regimens with or without added Carboplatin and showed higher pCR rates with inclusion of the platinum-based agent. The CALGB 40603/Alliance trial assessed the value of adding Bevacizumab +/− Carboplatin to neoadjuvant chemotherapy in stage II and III TNBC in 443 patients [24]. The proportion of patients who attained pCR increased remarkably from 41% to 54% with the use of Carboplatin (OR = 1.71; *p* = 0.0029). The long-term OS was not powered in the trial, and the addition of Carboplatin to conventional chemotherapy did not increase long-term OS [25]. The GeparSixto trial involved 595 patients diagnosed with stages II or III TNBC, who were randomized to receive either Carboplatin or no Carboplatin with a backbone regimen of Paclitaxel, liposomal Doxorubicin, and Bevacizumab [26]. The pCR rates were considerably higher in the carboplatin group: 53.2% vs. 36.9 (*p* = 0.005) (Table 2). The result of a meta-analysis looking at 9 randomized controlled trials (RCTs) (*n* = 2109) revealed that adding platinum to neoadjuvant chemotherapy considerably improved pCR rate from 37.0% to 52.1% (OR 1.96, 95% confidential interval (CI) 1.46–2.62, *p* < 0.001) [27]. Loibl et al. presented their updates from the BrighTNess trial, a randomized phase III clinical trial, with three treatment arms and a total of 634 patients with TNBC: the established neoadjuvant regimen consisting of Paclitaxel alone (P) (*n* = 158), Paclitaxel and Carboplatin alone (PCb) (*n* = 160), and Paclitaxel, Carboplatin and the PPAR inhibitor Veliparib (PCbV) (*n* = 316). Event-free survival, OS, and safety outcomes were assessed with a ≥4 years of follow-up period [28]. pCR was significantly improved when Carboplatin was added, with or without the addition of Veliparib, to Paclitaxel-based neoadjuvant chemotherapy. Also, adding Carboplatin to Paclitaxel improved pCR and EFS without increasing myelodysplastic syndrome or acute myeloid leukemia [28]. When compared to P alone, HR for EFS with PCbV was 0.63 (95% CI: 0.43–0.92, *p* = 0.016), and HR for EFS with PCb was 0.57 (95% CI 0.36–0.91, *p* = 0.018) [28]. Based on the latest American Society of Oncology (ASCO) recommendations, carboplatin may be offered to patients with TNBC to increase pathologic complete response [29].

## 4. Detecting PDL-1 Expression in TNBC

As the importance of immunotherapies targeting PD-1/ PD-L1 is evolving, concerns are arising regarding diagnostic tests that detect the level of these molecules and thus predict outcomes in cancer patients. Routinely, immunohistochemistry is used to measure PD-L1 expression. Currently, many of the commercially available tests are designed by antibody clones that detect the presence of the PD-L1 protein. Moreover, multiple expression scores and cutoffs exist [33]. Of the relevant PD-L1 scores are the tumor cell score, tumor proportion score, the immune cell score, and the combined positive score [34,35].

There are four PD-L1 immunohistochemical (IHC) assays registered with the FDA, using four different PD-L1 antibodies (22C3, 28–8, SP263, SP142), on two different IHC platforms (Dako and Ventana), each with their own scoring systems [36]. Attempts at harmonization of PD-L1 IHC antibodies and staining platforms are underway [37]. While PD-L1 IHC can be used to predict likelihood of response to anti-PD-1 or anti-PD-L1 therapy, a proportion of patients that are negative can have response and identification of alternative biomarkers is critical to further refine selection of patients most likely to respond to these therapies [36,38,39,40].

## 5. Beyond Chemotherapy for Metastatic Triple Negative Breast Cancer

### 5.1. Antibody Drug Conjugates-Sacituzumab Govitecan

Given the lack of defined target for drug development in TNBC, and the need to improve prognosis of this disease, several effective therapies have been identified [41]. Antibody-drug conjugates (ADCs) are combinations of a monoclonal antibody and a cytotoxic drug and have been used in many instances to treat other tumors: trastuzumab emtansine for metastatic HER2 + BC, and brentuximab vedotin for the treatment of recurrent Hodgkin lymphoma and anaplastic large cell lymphoma [41,42,43].

Sacituzumab govitecan (SG) is a novel ADC composed of the anti-trophoblast cell-surface antigen 2 (Trop-2) linked to the antineoplastic drug SN-38, which is the active metabolite of Irinotecan (a Topoisomerase I inhibitor) [44,45]. Trop-2 was found to be upregulated in all cancer types, particularly breast, colon, prostate, pancreatic, and lung cancers, making it an ideal potential therapeutic target [41,46,47]. It allows for targeted delivery of SN-38 to tumor cells, by bypassing the pharmacokinetic issues seen with Irinotecan [41,44,45]

**Figure 2:**
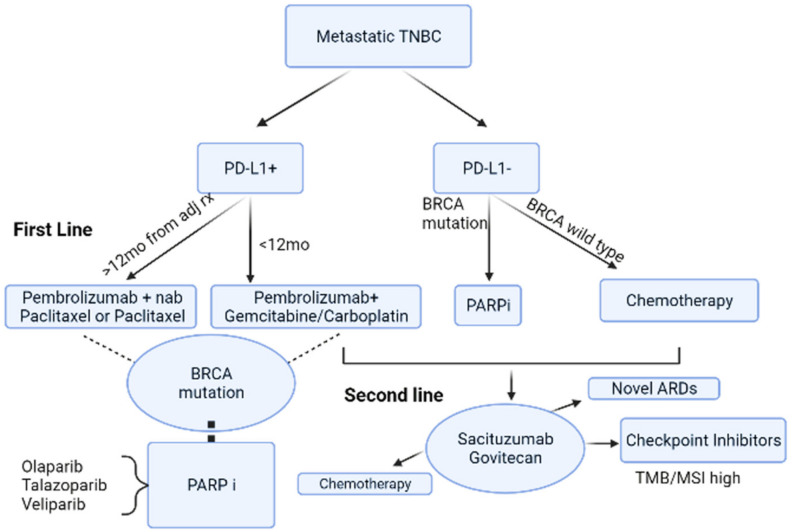
Treatment of metastatic triple negative breast cancer. PARP = Poly (ADP-ribose) polymerase PD-L1 = Programmed death-ligand 1 TMB = tumor mutational burden MSI = microsatellite instability. In general, the first line treatment for mTNBC depends on the PDL-1 status. If the status is confirmed to be PD-L1 +ve, then the patient is treated with Pembrolizumab along with a second agent that is determined by the duration of treatment (12 months cutoff) from adjuvant chemotherapy. If the status is confirmed to be PD-L1 −ve, then the patient is treated with chemotherapy if no BRCA mutation is detected. PARPi inhibitors can be used when a BRCA mutation is detected irrespective of PD-L1 status. The second line of treatment is Sacituzumab Govitecan or other novel ADCs or checkpoint inhibitors.

The drug was first approved by the United States Food and Drug Administration (FDA) in April 2020 after showing an Objective Response Rate (ORR) of 33%, a median progression free survival (PFS) of 5.5 months and an overall survival (OS) of 13 months in a phase 1–2 single-group basket trial (IMMU-132-01) [44]. It received its final approval after the ASCENT trial, a phase III study which evaluated the efficacy and safety of SG as compared to standard of care chemotherapy based on physicians’ choice (Eribulin, Vinorelbine, Capecitabine, or Gemcitabine) for the treatment of relapsed or refractory TNBC [44]. Patients were randomly assigned to receive a dose of 10 mg per kilogram of body weight of SG on days 1 and 8 of each 21-day cycle or a single chemotherapeutic agent as per standard of care [44,45]. Efficacy of SG in the full population was then reported as follows: patients on SG had an improved median PFS (4.8 months vs. 1.7 month) and better OS (11.8 months, 95% CI: 10.5–13.8 vs. 6.9 months, 95% CI: 5.9–7.7) as compared to patients who received chemotherapy [44,45], showing a significant benefit of SG over chemotherapy. The side effects reported in both groups were similar: neutropenia, diarrhea, nausea, alopecia, fatigue, and anemia [45]. Severe adverse events leading to withdrawal from the study occurred in 5% of patients assigned to each group and no deaths were attributed to treatment with SG compared to one deemed related to chemotherapy [44]. However, the *Ascent* trial had some limitations. First, 32 patients withdrew consent from the chemotherapy group before the trial’s initiation. Second, SG was compared to multiple chemotherapeutic drugs, each with its own safety profile. Last, patients were not mandated to undergo biopsies to confirm their diagnosis before entering the trial [44]. To wrap up, Sacituzumab Govitecan has been approved by the FDA in April 2021 after the results of the Ascent trial (ASCENT; NCT02574455) since the PFS and OS were significantly longer with Sacituzumab Govitecan than with single-agent chemotherapy among patients with metastatic triple-negative BC [48]. The recommended sacituzumab govitecan dose is 10 mg/kg once weekly on days 1 and 8 of 21-day treatment cycles until disease progression or unacceptable toxicity [49]. To note, Figure 2 depicts the first and second line of therapy for mTNBC. The rest of the manuscript will be dealing with other lines of treatments used in mTNBC in more details.

### 5.2. Immune Check-Point Inhibitors

Immune check-point inhibitors, such as programmed cell death protein 1 (PD-1) and its ligand PD-L1, have become important novel therapeutic targets that modulate T-cell activation and suppress tumor growth [50]. It has been recently confirmed that certain TNBCs displays high PD-L1 expression compared to other BC subtypes, linked to remarkable genomic instability, and increased immune infiltration [50,51]. These properties confer patients with TNBC as good candidates for immunotherapy with PD-L1 inhibitors such as atezolizumab or pembrolizumab [50].

#### 5.2.1. Atezolizumab

Atezolizumab is a humanized immunoglobulin G1 (IgG1) monoclonal antibody. It targets PD-L1 on tumor cells, inhibiting its binding to its receptors PD-1 or CD80 on the surface of T-cells [50]. As the binding of PD-L1 to PD-1 or CD-80 inhibits the activation of T-lymphocytes, this blockade impairs the tumor immune system evasion by allowing T-cell responses to occur, thus enhancing anti-tumor activity [50]. Several trials have been conducted to assess the efficacy and safety of atezolizumab in the treatment of TNBC, eventually leading to its approval for metastatic TNBC in 2019. This is the first instance of approval of immune checkpoint inhibitor therapy in the treatment of BC.

The *IMpassion 130* trial compared the results of treatment with atezolizumab plus nab-paclitaxel (A + nP) to placebo plus nab-paclitaxel (P + nP) in 902 patients with de novo metastatic TNBC, who were stratified according to PD-L1 status. The nanoparticle albumin-bound (nab) was added to paclitaxel to avoid the use of glucocorticoids that were thought to affect immunotherapy activity [52]. An improved OS at the end of an 18.8-month follow-up period, with a median increase of 7.5 months, was observed in the PD- L1 +ve population who received A + nP The OS was reported to be 25.4 months in the atezolizumab PD-L1 +ve group vs. 17.9 months in the placebo PD-L1 +ve group, but could not be tested formally due to statistical considerations [52,53]. A longer PFS was also noted in both the intention to treat population and the PD-L1 +ve subgroup of patients [52,54]. The FDA based its accelerated approval of Tecentriq, atezolizumab, on results from the *IMpassion 130* trial.

Adverse events reported were consistent with the established toxicity profile of every agent. Conversely, the incidence of grade 3 or 4 adverse events, such as neutropenia, febrile neutropenia, and hypertension, was higher in the A + nP group as compared to the P + nP group (48.7% vs. 42.2%) [50,52,55]. Serious adverse events were also more commonly associated with the treatment group (22.8% vs. 18.3%). Immune-related hypothyroidism, although rare, occurred at a rate of 17.3% in the treatment group compared to 4.3% in the placebo group [52]. Accelerated approval by the FDA and European Medicines Agency of treatment with A + nP for patients with metastatic or locally advanced and unresectable PD-L1 +ve TNBC was eventually granted [52].

Later, the IMpassion 131 trial evaluated atezolizumab and paclitaxel without being coupled to nab, as first-line treatment for advanced TNBC [51]. However, the primary objective was not met as this combination did not significantly prolong neither PFS in patients with PD-L1 +ve aTNBC nor overall survival [51].The use of steroids instead of nab might potentially have dampened the effects of immunotherapy, but this was not confirmed and the reasons for this difference were not defined [51].

Given that the follow-up period for the IMpassion 130 trial was only about a year, the FDA decided to base the continued approval of atezolizumab in BC on the IMpassion 131 trial. Taking into consideration the results of the latter, the FDA voluntarily withdrew the use of atezolizumab in metastatic TNBC.

The IMpassion 031 trial evaluated the efficacy and safety of atezolizumab as a neoadjuvant therapy vs. placebo combined with nab-paclitaxel in early-stage TNBC. Both regimens were followed by doxorubicin plus cyclophosphamide [56]. This randomized trial depicted a significant improved pathological complete response rate in the atezolizumab plus chemotherapy group compared to the placebo plus chemotherapy group, regardless of PD-L1 status, disease stage or lymph node status [56]. This result contrasts with finding from the IMpassion 130 study in which atezolizumab was more beneficial in patients with PD-L1 + ve TNBC. It is worth noting that this trial also reported an acceptable safety profile of the regimen used.

Last, the predictive value of gene-expression profiles (GEPs) and their dynamics during therapy has recently been assessed in the *NeoTRIPaPDL1 trial*. Patients with TNBC (*n* = 258) either received eight cycles of nab-paclitaxel/carbo (CT) with/without atezolizumab (CTA) [57]. RNA-sequencing was performed pre-treatment along with day 1 of cycle 2 on 242 patients (93.8%) and 161 patients (62.4%) respectively [57]. Pre-treatment, binary IO score was predictive of pCR in CTA with an OR 3.64 with 95% CI of 1.68–7.90 (*p* = 0.001), but not in the CT arm where the OR was 1.31 with a 95% CI of 0.64–2.67 (*p* = 0.046). The BL1 subtype had the highest pCR rate (CTA 70.3%, CT 54.3%) while the LAR subtype had the lowest pCR rate (CTA 22.2%, CT 18.8%) [57]. Interestingly, high angiogenesis and fatty acid/cholesterol were independently linked to resistance in CTA (*p* = 0.005), but not CT (*p* = 0.02). Glutamine metabolism was linked to resistance in the CTA arm only [57]. This suggests new potential therapeutic targets. Super-responders in the CTA arm had high expression of some immune signatures. A CD8 above the median was associated with 58.6% and 61.7% pCR rate in CTA and CT arm, respectively. Additional predictive information could be obtained from combining both baseline values and dynamic values of some biomarkers instead of each alone [57].

On March 2019, the FDA approved atezolizumab use in addition to nab-paclitaxel in the treatment of PD-L1 positive, unresectable locally advanced or mTNBC in the following dose: 840 mg intravenous infusion over 60 min, followed by 100 mg/m^2^ paclitaxel protein-bound [58]. However, accelerated approval was not maintained due to a change in treatment landscape [59].

#### 5.2.2. Pembrolizumab

Pembrolizumab is a humanized monoclonal immunoglobulin (IgG4) antibody against PD-1 that blocks the interaction between the receptor and its ligand leading to a potent antitumor activity [60]. When combined with chemotherapy, it has a promising anti-tumor activity without increase in toxicity [60]. *The Keynote-355 trial* evaluated the efficacy and safety of pembrolizumab plus chemotherapy compared to placebo plus chemotherapy as a first line therapy in patients with untreated locally recurrent unresectable TNBC or metastatic TNBC [61]. The use of Pembrolizumab in addition to chemotherapy resulted in a remarkable prolongation in PFS compared to chemotherapy alone: 9.7 months in the pembrolizumab group vs. 5.6 months in the placebo group [61]. However, a greater response was noted in patients with PD-L1 +ve tumors. Concerning the safety of the treatment, the combination of pembrolizumab and chemotherapy did not increase the rates of usual adverse events seen with chemotherapy [61]. Immune-mediated adverse events occurred more frequently in patients taking pembrolizumab (5%) compared to chemotherapy alone (0%) as expected [61]. The results of this trial were presented after a median follow up of 44.1 months, as of June 2021 [62,63]. An improved overall survival was noted in the pembrolizumab and chemotherapy group with a median OS of 23.0 months (95% CI 19.0–26.3) compared to chemotherapy alone (15.5 months [95% CI 13.9–17.2]), validating results reported in the prior interim analysis of Keynote-355 [62]. This statistically significant improvement in OS proves the benefit of the addition of pembrolizumab to chemotherapy in the treatment of patients with previously untreated and inoperable or metastatic TNBC with tumors expressing PD-L1 [62].

The *Keynote-522* trial evaluated the efficacy and safety of neoadjuvant pembrolizumab-chemotherapy vs. neoadjuvant placebo-chemotherapy, in patients with early TNBC [64]. In this trial, the percentage of patients who responded to treatment was significantly higher in the pembrolizumab-chemotherapy group (64.8%) compared to placebo-chemotherapy group (51.2%) [64]. The efficacy of pembrolizumab was shown to be consistent regardless of PD-L1 status [64]. Moreover, the hazard ratio for disease progression, local or distant recurrence, having a second primary tumor, or death were in favor of treatment with pembrolizumab-chemotherapy; with distant recurrence being the most common event [64]. Safety risks were as expected regarding each regimen’s profiles [64]. The treatment protocol was reviewed by the FDA, and the Oncologic Drug Advisory Committee (ODAC) meeting that deferred its accelerated approval.

The Targeted Agent and Profiling Utilization Registry (TAPUR) study is a prospective phase II trial that evaluated the antitumor activity of commercially available targeted agents beyond their approved indications [60,65]. It reports results of patients with metastatic BC with high tumor burden (HTMB) treated with pembrolizumab as a single agent [60]. Reporting disease control (DC), which is defined as either complete or partial response to treatment at 8 weeks or later, or a stable disease (SD) of at least 16 weeks, was the study’s primary endpoint [60]. 28 female patients, with metastatic BC with HTMB ranging from 9 to 37 Mut/Mb, were enrolled. 89% had HER2-negative tumors and 46% had TNBC [60]. Disease control was seen in 10 of 28 patients at a rate of 37% (95% CI, 21–50) while the objective response OR rate was 21% (95% CI, 8–41) [60]. The reported median PFS was 10.6 week (95% CI, 7.7 to 21.1 weeks) and the median OS was 30.6 weeks (95% CI < 18.3 to 103.3 weeks) [60]. No relationship was reported between the PFS and the TMB. Here it is worth noting that the study was not powered to detect such an association in the first place [60]. Drug related adverse effects of grade 3–4, such as pulmonary embolism, weight loss, hypoalbuminemia, and hyponatremia, were reported in 11% of patients, and these were considered possibly related to pembrolizumab along with other events [60]. These data support the FDA’s approval of pembrolizumab in the treatment of patients with unresectable or metastatic solid tumors with HTMB of 10 Mut/Mb or more [60]. This study was not able to identify the population of patients with BC that would benefit the most from this treatment modality because of the relatively small size of study population, its heterogeneity, and limited data collection [60]. However, it was able to demonstrate that the single agent pembrolizumab has a remarkable activity in pretreated patients with metastatic BC with HTMB [60].

During ODAC’s meeting in 27–29 April 2021, updates were given on 6 accelerated approvals for immune checkpoint inhibitors [66]. Atezolizumab in combination with nab-paclitaxel for locally advanced/ metastatic TNBC expressing PD-L1 was approved [66]. Atezolizumab was also approved for locally advanced/metastatic urothelial carcinoma [66]. Pembrolizumab was accepted for locally advanced or metastatic urothelial carcinoma as well as gastric or GE junction adenocarcinoma and hepatocellular carcinoma [66]. It was also FDA approved for high-risk, early-stage, TNBC, locally unresectable or metastatic and expressing PD-L1, at a dose of 200 mg in addition to 80 mg of paclitaxel [67].

### 5.3. Poly-Adenosine Diphosphate Ribose (ADP) Polymerase Inhibitors (PARPi)

PARP is a single stranded DNA- Breaks (SSBs) repair protein that plays an important role in the initiation of the SSBs repair by DNA base excision [68]. BRCA1/2 are essential in the repair of double stranded DNA breaks (DSBs) by the process of homologous recombination (HR) [68]. When PARP is inhibited, SSBs accumulate in cells, leading to DSBs. Cells that are also BRCA1/2 mutant will ultimately die because they lack the ability to repair DSBs; this is referred to as synthetic lethality [68]. This is the rationale behind the use of PARPis in the treatment of patients with BRCA1/2 BC. The FDA has approved the use of olaparib and talazoparib in metastatic BC and many emerging studies are evaluating the potential role of PARPis in “BRCA-like” TNBC with HR deficiency.

#### 5.3.1. Olaparib

The PARPi olaparib has been approved for the treatment of patients with recurrent ovarian cancer who have a concurrent BRCA mutation [69,70]. It has also shown potential benefits for patients with metastatic BC and a germline BRCA mutation [71]. The OlympiAD trial evaluated the efficacy and safety of olaparib compared to standard chemotherapy among patients with HER2 negative and a germline BRCA mutation [69]. 302 patients were randomly assigned to receive either Olaparib tablets (300 mg twice a day) or standard chemotherapy (capecitabine, eribulin mesylate or vinorelbine) [69]. The study met its primary endpoint with a significantly longer median PFS noted in patients taking Olaparib (7 months) compared to standard treatment (4.2 months) (hazard ratio for disease progression or death, 0.58, 95% confidence interval [CI], 0.43 to 0.80; *p* < 0.001) [69]. OS, however, did not differ significantly. Concerning the response to treatment, 59.9% of patients taking olaparib responded with 9% complete response whereas 28.8% of patients taking standard treatment responded, out of which only 1.5% had a complete response [69]. Disease progression was also noted to be 42% lower with olaparib. Concerning safety risks, grade 3 adverse events were noted at a higher rate in the standard treatment group (50.5%) compared to the olaparib group (36.6%) [69]. Leukopenia, dyspnea, and thrombocytopenia are grade 3 adverse events that occurred in 2% of patient of group [69].

Recently, Tutt et al. assessed the use of Olaparib for patients with non-metastatic BRCA mutated BC with residual disease after completion of local treatment and neoadjuvant or adjuvant chemotherapy. A total of 1836 patients were followed up to a median of 2.5 years. The 3-year invasive DFS observed within the Olaparib group 85.9%, significantly higher than 77.1% in the placebo group (difference, 8.8 % points; 95% CI, 4.5 to 13.0; HR for invasive disease or death, 0.58; 99.5% CI, 0.41 to 0.82; *p* < 0.001) [72]. Olaparib use was also associated with a superior DFS (87.5% vs. 80.4%; *p* < 0.001) [72]. The FDA granted priority review to Olaparib for adjuvant treatment BRCA-mutated Her2-ve high risk early BC on 30 November 2021. A summary of information on Olaparib and other newly approved non-chemotherapy anti-TNBC agents can be found in Table 3.

The phase 3 *EMBRACA* trial evaluated the efficacy and safety of talazoparib compared to standard treatment with a chemotherapeutic agent of the physician’s choice in patients with locally advanced or metastatic BC with germline BRCA1/2 mutation [73]. 431 patients were randomly assigned in a 2:1 ration to either talazoparib group (1 mg orally once daily) or standard therapy group (capecitabine, eribulin, gemcitabine, or vinorelbine) [73]. The primary endpoint was radiologic PFS, and it was reported to be longer among patients taking yalazoparib (8.6 months) as compared to standard chemotherapy (5.6 months) [73]. At the interim analysis, patients in the yalazoparib group were noted to have an OS of 22.3 months compared to 19.5 months for patients taking standard chemotherapy [73]. Moreover, 5.5% of patients treated with yalazoparib noted a complete response whereas none did in the standard treatment group [73]. Primary and secondary endpoints favored talazoparib over standard chemotherapy. The adverse events reported in the talazoparib group were nausea, anemia, and fatigue. More hematologic and less hematologic adverse events occurred with talazoparib than with standard therapy [73]. Patient-reported outcomes were in favor of talazoparib use and indicated that it had a good side-effect profile [73].

Many genes other than BRCA1/2 are implicated in DNA repair. For instance, studies in prostate cancer suggested that PARPis may be beneficial in patients with mutations in HR-related genes, beyond RBCA1/2. Moreover, it has been shown in previous studies on ovarian cancer that patients with somatic BRCA1/2 mutations may also benefit from treatment with a PARPi [74]. Accordingly, the Olaparib Expanded (Translational Breast Cancer Research Consortium) TBCRC 048 trial was conducted to assess whether patients with metastatic BC with germline mutation in HR-related genes other than BRCA1/2 or with somatic BRCA1/2 mutations could benefit from treatment with the PARPi olaparib [75]. The patients included in this study had either somatic BRCA1/2 mutation or one of the following germline mutations: ATM, ATR, BAP1, BARD1, BLM, BRIP1, CHEK1, CHEK2, CDK12, FANCA, FANCC, FANCD2, FANCF, MRE11A, NBN, PALB2, RAD50, RAD51C, RAD51D, or WRN [75,76]. 55 patients were split into two cohorts: patients with germline mutation in HR-related genes vs. patients with somatic mutation in BRCA1/2 and the aforementioned genes [75]. The treatment consisted of 300 mg of olaparib administered orally twice a day until any of the following occurred: disease progression, unacceptable toxicity, or withdrawal of consent [75]. In cohort 1, the ORR was 33%, with all responses noted in patients harboring the gPABL2 mutation, making the ORR of patients with gPABL2 as high as 82% [75]. In cohort 2 (somatic mutations in HR- related genes), the ORR was 31% and all responses were seen in patients having somatic BRCA1/2 mutations. The ORR of patients with somatic BRCA1/2 mutations was 50%, and the median PFS was 6.3 months vs. 13.3 months in cohort 1 [75].

According to this study, it is important to perform germline and tumor genomic profiling in patients with metastatic BC to detect suitable candidates for treatment with PARPis.

#### 5.3.2. Veliparib

In order to further support the aforementioned conclusion, additional trials using the PARPi Veliparib, which harbors a different off-target kinase pharmacology when compared to Olaparib [78], were conducted. The SWOG S1416 trial was conducted to compare outcomes in patients with different tumor genomic characteristics treated with cisplatin with or without veliparib [79]. 335 patients were recruited having TNBC or BRCA1/2 +ve metastatic BC and were divided into three groups: patients with germline BRCA mutation, patients with BRCA-like mutation in HR-genes and non-BRCA-like mutation [79]. No statistically significant difference in PFS was noted in the group of patients having a germline BRCA mutation between veliparib therapy and placebo. In the BRCA-like group, PFS with veliparib treatment was prolonged compared to placebo, 5.7 vs. 4.3 months (HR = 0.58; *p* = 0.023, 1 years PFS 20% vs. 7%) [79]. OS and ORR were also better with veliparib treatment in this same group. The non-BRCA like group of patients and the unclassified one did not benefit of veliparib as there was no significant difference in PFS [79]. Concerning toxicities, grade 3–4 neutropenia and anemia occurred at a higher rate with veliparib treatment than with placebo [79]. Moreover, in BROCADE3, a phase III randomized trial, the authors assigned patients with a BRCA1 or BRCA2 mutation as follows: 337 patients were given Veliparib plus carboplatin-paclitaxel versus 172 patients who received placebo plus carboplatin-paclitaxel [80]. They report a median PFS of 14.5 months in the Veliparib group compared to 12.6 months in the placebo group (HR = 0.71). They went on to discuss that when Veliparib was added to the treatment plan alongside carboplatin-paclitaxel or as monotherapy in case the latter were discontinued, PFS improved remarkably by year 2 and 3 after trial initiation [81].

Accordingly, patients with BRCA-like mutations benefit the most from the addition of Veliparib to cisplatin and therefore, further combinations of platinum agents plus PARPis should be investigated [79].

### 5.4. Androgen Receptor Targeted Agents

Hormone receptor negative BC generally does not respond to endocrine-targeted therapies. In a previous study done at Memorial Sloan-Kettering Cancer Center, a subset of estrogen/progesterone receptor (ER/PrG)-negative cancers were identified and shown to have a transcriptional profile like the molecular apocrine or luminal androgen receptor AR [82,83]. Accordingly, the hypothesis that AR inhibition would potentially benefit patients with AR +ve ER/PgR negative advanced BC was tested using different therapies and the results are listed below.

#### 5.4.1. Bicalutamide

Bicalutamide is a non-steroidal AR antagonist approved by the FDA in combination with luteinizing hormone-releasing hormone (LHRH) in the treatment of metastatic prostate cancer [84]. A multicenter phase II trial was conducted to evaluate the efficacy Bicalutamide in ER/PgR negative metastatic BC. Patients enrolled in the study were administered 150 mg of Bicalutamide orally every day and they were treated until disease progression or intolerable side effects were observed [84]. The patient population enrolled in this trial represents TNBC. Furthermore, in the subset of patients with AR +ve ER/PgR negative metastatic BC, a clinical benefit rate (CBR) of 19% was noted, realizing this study’s endpoint [84]. This treatment was well-tolerated, and the adverse events reported included fatigue, hot flashes, limb edema, and transaminase elevation [84]. This study supports the hypothesis that targeting AR could benefit patients with ER/PgR negative metastatic BC.

#### 5.4.2. Abiraterone

Abiraterone Acetate (AA) is a selective, irreversible, and potent inhibitor of 17-[α]-hydroxylase/17,20-lyase (CYP17) that is commonly used in prostate cancer resistant to castration [85,86]. A study was designed to evaluate the benefit of AA in addition to prednisone in the treatment of AR +ve TNBC [87]. The primary endpoint of this phase II trial was to reach a CBR of 25% at 6 months. However, the CBR at 6 months was 20%, so the study did not meet its aim [87]. Concerning the secondary outcomes, ORR was 6.7%, and the median PFS was 2.8 months [87]. As for the safety of this treatment, the most common drug associated side effects were fatigue (17.6%), hypertension (11.8%), hypokalemia (8.8%) and nausea (5.9%), all of which were managed easily [87]. Even though statistically insignificant, this study demonstrated a clinical benefit of treatment with AA and prednisone in some patients with molecular apocrine-like tumors [87].

#### 5.4.3. Enzalutamide

Enzalutamide is an AR inhibitor with activity on multiple steps in AR signaling pathway [88]. It has been approved for the treatment of castration resistant prostate cancer [89,90]. The results of a phase II trial that evaluated the benefit of Enzalutamide in patients with TBC and AR +ve are reported [88]. 118 patients were enrolled of which 78 were included in the evaluable group, and they received 160mg of Enzalutamide one daily until disease progression [88]. This study met its primary endpoint given that 25% of the intention to treat patients and 33% of the evaluable patients achieved a CBR at 16 weeks. Moreover, 20% of the ITT patients and 28% of the evaluable ones achieved a CBR at 24 weeks [88]. The median PFS was 2.9 months in the ITT population and 3.3 months in the evaluable subpopulation [88]. Median OS was 12.7 months and 17.6 months in the ITT and the evaluable subgroup respectively [88]. Results from this study support findings of other trials that highlighted the benefit of AR- targeted therapy in patients with TNBC. Most of the findings presented in this section are summarized in Table 4.

## 6. Conclusions

The treatment of TNBC is evolving significantly. Defining the specific tumor molecular profile including PDL-1 and androgen receptor testing is expanding treatment options in the clinical setting. Identifying more targetable, novel biomarkers that may better define therapeutic targets or prognostic markers is currently underway. In the face of such innovative therapies, TNBC nomenclature is expected to be updated in favor of other terms which would help direct therapy, and further redefine TNBC’s heterogeneity. More clinical trials are needed to provide additional support to the efficacy of new drugs and to the relevance of identifying the targeted biomarkers.

## Figures and Tables

**Table 1:** TNBC subtypes based on the FUSCC (Fudan University Shanghai Cancer Center) classification criteria [6].

FUSCC Classification	Pathways	
IM (immunomodulatory)	↑	Cytokine–cytokine receptor interaction T cell receptor signaling pathway B cell receptor signaling pathway Chemokine signaling pathway NF-kappa-B signaling pathway
LAR (luminal androgen receptor)	↑	Steroid hormone biosynthesis Porphyrin and chlorophyll metabolism PPAR signaling pathway Androgen and estrogen metabolism
MES (mesenchymal-like)	↑	ECM-receptor interaction Focal adhesion TGF-beta signaling pathway ABC transporter Adipocytokine signaling pathway
BLIS (basal-like and immune suppressed)	↑	Mitotic cell cycle Mitotic prometaphase M phase of mitotic cell cycle DNA replication DNA repair
	↓	Immune response Innate immune response T cell receptor signaling

**Table 2:** Frequencies of pCR from clinical trials involving carboplatin.

Trials (References)	Regimen 1 (R1)	Nb. of Patients	Regimen 2 (R2)	Nb of Patients	pCR Rate (R1 vs. R2)	*p*-Value
GeparOcto GBG84 [30]	**P** + **NPLD** + **Cb**; q1w for 18 weeks	203	**E** then **P** then **C**; q2w/3 cycles over 18 weeks	200	51.7% vs. 48.5%	0.518
GALGB40603 Alliance [24]	(**P** q1w for 12 weeks then **ddAC** q2w/4 cycles) + (**Cb** q3w/4 cycles ± **Bev**. q2w/9cycles)	221	**P** q1w for 12 weeks then **ddAC** q2w/4 cycle	212	54% vs. 41%	0.0029
GeparSixto GBG66 [26]	(P q1w for 18 weeks + NPLD q1w for 18 weeks + Bev. q3w/6 cycles) + Cb q1w for 18 weeks	158	**P** q1w for 18 weeks + **NPLD** q1w for 18 weeks + **Bev**. q3w/6 cycles	157	53.2% vs. 36.9%	0.005
Zhang et al. [31]	**P** + **Cb** q3w/4–6 cycles	47	**P** + **E** q3w/4–6 cycles	44	38.6% vs. 14.0%	0.014
Ando et al. [32]	(**P** q2w/2 cycles then **CEF** q2w/4 cycles) + **Cb** q3w/4 cycles	37	**P** q2w/2 cycles then **CEF** q2w/4 cycles	38	61.2% vs. 6.3%	0.003

P = Paclitaxel; NPLD = Nonpegylated Liposomal Doxorubicin; Cb = Carboplatin; E = Epirubicin; C = Cyclophosphamide; ddAC = Doxorubicin plus Cyclophosphamide; Bev. = Bevacizumab; CEF = Cyclophosphamide plus Epirubicin plus 5-fluorouracil.

**Table 3:** Summary of newly approved non-chemotherapy anti-TNBC agents.

Trials (References)	Drug & Approval Date	Indication/Inclusion Criteria	Dosage
OlympiA [72]	Olaparib November 2021	BRCA 1/2-mutated HER2-negative BCPrevious recipients of adjuvant or neoadjuvant chemotherapy ±RT (definitive local therapy)Pathologically confirmed residual disease post-operatively Previous recipients of adjuvant or neoadjuvant chemotherapy ±RT (definitive local therapy)Pathologically confirmed residual disease post-operatively Pathologically confirmed residual disease post-operatively	300 mg PO cycled q28days/1 year twice daily ± food
KEYNOTE-522 [64]	Pembrolizumab + Chemotherapy July 2021	Non-metastatic TNBC Tumors 1–2 cm + 1–9 LN involved or tumors ≥2cm ± 1–9 LN involved Regardless of PD-L1 status	200 mg IV q21 days/8 cycles + chemotherapy pre-operatively followed by 200 mg IV q21 days/9 cycles as single agent post-operatively
IMMU-132-01 [77]	Sacituzumab govitecan-hziy April 2021	Metastatic TNBC No brain metastasis present Recurrent disease after 2 completed lines of therapy	10 mg/kg on days 1 & 8 q21 days IV until disease progression/unacceptable adverse events
IMpassion130 [52]	Atezolizumab + nab-paclitaxel April 2019	Metastatic TNBC No prior chemotherapy or targeted systemic therapy for metastatic disease RT or neo-adjuvant chemotherapy completed ≥12 months allowed	Atezolizumab 840 mg IV day 1 & 15 + 100 mg/m2 on day 1, 8 & 15 nab-paclitaxel q28 days/6 cycle or until disease progression/unacceptable adverse events

**Table 4:** Targeting TNBC Beyond Chemotherapy.

Regimen	Summary	Primary Endpoint
Antibody Drug Conjugates		
Sacituzumab Govitecan (SG) [44]	Relapsed metastatic TNBC or metastatic TNBC refractory to ≥2 lines of treatment	ASCENT: Improved median PFS with SG (4.8 months) compared to SOC chemotherapy (1.7 months) (*p* < 0.001)
Immune-checkpoint inhibitors		
Atezolizumab [46,50,51,52,53,54,55,56,66]	Approved for use with Nab- paclitaxel (nP) Metastatic PD-L1 positive TNBC or locally advanced and unresectable PD-L1 positive TNBC. Locally advanced/metastatic urothelial carcinoma.	IMpassion130: 9.5 months increase in OS with Atezolizumab + nP compared to Placebo + nP (*p* = 0.08) IMpassion131: no significantly improved in PFS with Atezolizumab + paclitaxel without nab vs. placebo + paclitaxel (*p* = 0.20) IMpassion031: improved pCR with Atezolizumab + chemotherapy vs. placebo + chemotherapy (Rate difference: 17%) (*p* = 0·0044)
Pembrolizumab [60,61,64]	Untreated and unresectable, locally recurrent TNBC or metastatic TNBC Also approved for: locally advanced or metastatic urothelial carcinoma, gastric adenocarcinoma, GE junction adenocarcinoma and hepatocellular carcinoma.	Keynote-355: Prolonged PFS with pembrolizumab + chemotherapy (9.7 months) vs. chemotherapy alone (5.6 months) (*p* = 0.0012) Keynote-522: Better pCR with Pembrolizumab + chemotherapy (64.8%) compared to placebo + chemotherapy (51.2%) (*p* < 0.001) TAPUR: Stable disease for ≥16 weeks was achieved in 37% with Pembrolizumab monotherapy in high-tumor burden disease
Poly Adenosine diphosphate-ribose PARP inhibitors		
Olaparib [69,70,71,75]	Also approved for: recurrent ovarian cancer with BRCA mutation Deleterious germline BRCA mutated, HER2- metastatic BC previously treated with chemotherapy	OlympiAD: Prolonged PFS with Olaparib (7.0 months) compared to standard treatment (4.2 months) (*p* < 0.001) TBCRC 048: Significant Objective Response Rate (ORR) seen with germline PABL2 (ORR: 82%) and somatic BRCA1/2 (ORR: 50%) Olympia: Improved 3-year invasive DFS with olaparib (85.9%) as compared to placebo (77.1%) (*p* < 0.001)
Talazoparib [73,91]	Locally advanced Her2- BC not-amenable to curative therapy or metastatic Her2- BC With germline BRCA1/2 mutation CNS metastasis eligible only if stable	EMBRACA: Prolonged median PFS and higher ORR with Talazoparib (PFS: 8.6 months; ORR: 62.6%) compared to SOC chemotherapy (5.6 months; ORR: 27.2%) (*p* < 0.001)
Veliparib [79]	Metastatic TNBC or germline mutated BRCA1/2-associated metastatic BC Had received <1 line of prior therapy	SWOG S1416: Improved PFS with Veliparib (5.7 months) compared to placebo (4.3 months) (*p* = 0.023) in BRCA-like mutation group, but not germline mutated BRCA group
Androgen receptor targeted agents		
Bicalutamide [86]	Unresectable locally advanced or metastatic BC ER/PgR-negative regardless of HER2 status, androgen-receptor positive Also approved for: metastatic prostate cancer	Multicenter phase II trial: CBR of 19% (95% CI: 7%–39%) and PFS of 12 weeks (95% CI: 11–22 weeks) were observed
Abiraterone [85,86,87]	Investigated in metastatic TNBC or locally advanced TNBC, androgen-receptor positive Approved for: castration-resistant prostate cancer	Multicenter phase II trial: Primary endpoint of 25% CBR 6 months not met
Enzalutamide [88,89,90]	Investigated in metastatic TNBC or locally advanced TNBC, androgen-receptor positive Approved for: castration-resistant prostate cancer	Multicenter phase II trial: assessment of CBR at 16 weeks showed 25% of the intention to treat group and 33% of the evaluable patients

## Abbreviations

A	doxorubicin
ACT	adriamycin, cyclophosphamide, and paclitaxel (taxol)
AR	androgen receptor
AUC	area under the curve
BC	Breast Cancer
Bev	bevacizumab
BL1	basal-like 1
BL2	basal-like 2
BLIA	basal-like immune-activated
BLIS	basal-like immunosuppressed
C	cyclophosphamide
CBR	clinical benefit rate
DC	ductal carcinoma
dd	dose-dense
ddAC	Dose-Dense Adjuvant Doxorubicin and Cyclophosphamide
E	epirubicin
ECM	extracellular matrix
ER	estrogen receptor
FUSCC	Fudan University Shanghai Cancer Center
G	gemcitabine
GO	Gene Ontology
HER2	human epidermal growth factor receptor 2
IM	immunomodulatory
LAR	luminal androgen receptor
lncRNAs	long noncoding RNAs
M	mesenchymal
MES	mesenchymal-like subtype
MSL	mesenchymal stem-like
MUC1	cell-surface mucin
N/A	not applicable
nP	nab-Paclitaxel
NPLD	non-pegylated liposomal doxorubicin
NST	neoadjuvant systemic treatment
OS	overall survival
P	paclitaxel
pCR	pathological complete response
PDGFRα	platelet-derived growth factor receptor α
PR	progesterone receptor
RCTs	randomized controlled trials
RFS	relapse-free survival
RT	radiotherapy
T	docetaxel
TGF	transforming growth factor
TNBC	Triple-negative breast cancer
